# Radiographic and clinical outcome of tibial plateau fractures treated with bone allograft

**DOI:** 10.1007/s00402-022-04461-x

**Published:** 2022-05-10

**Authors:** Sarah Meiser, Rohit Arora, Johannes Petersen, Alexander Keiler, Michael C. Liebensteiner, Johannes Dominikus Pallua, Alexander Wurm

**Affiliations:** 1 Medical University of Innsbruck, Anichstrasse 35, 6020 Innsbruck, Austria; 2 Dept. of Orthopaedics and Traumatology, Medical University of Innsbruck, Anichstrasse 35, 6020 Innsbruck, Austria; 3 Dept. of Radiology, Medical University of Innsbruck, Anichstrasse 35, 6020 Innsbruck, Austria; 4 Klinikum Freising, Radiologie Klinikum Freising, Alois-Steinecker-Straße 18, 85354 Freising, Germany

**Keywords:** Tibial plateau fractures, Bone graft, Bone allograft, Quantitative computed tomography, Bone mineral density

## Abstract

**Background:**

To determine the clinical outcome of patients who had been treated with bone allografts during open reduction and internal fixation (ORIF) of tibial head fractures.

**Methods:**

Patients who suffered a medial, lateral, or bicondylar fracture of the tibial plateau and underwent surgical treatment by open reduction and internal fixation (ORIF) using human femoral head bone allografts were included. Patients were invited to provide information for the following: Western Ontario and McMaster Universities Osteoarthritis Index (WOMAC), EuroQol Five Dimension score (EQ-5D), Lower Extremity Functional Scale (LEFS) and Parker Mobility Score. Bone mineral density (BMD) of the allograft area and the healthy human bone tissue were measured by quantitative computed tomography.

**Results:**

A total of 22 patients with a mean follow-up time of 2.88 ± 2.46 years were included in our study. The most common fractures observed in this study were classified as Schatzker II (11 patients, 50.0%) or AO/OTA 41.B3 (12 patients, 54.5%) fractures. Postoperative WOMAC total was 13.0 (IQR = 16.3, range 0–33). Median quality of life (EQ-5D) score was 0.887 ± 0.121 (range 0.361–1.000). Median Lower Extremity Functional Scale (LEFS) score was 57.5 ± 19.0 (range 33–79). Mean Parker Mobility Score was 9 (range 6–9). Median bone mineral density (BMD) for the whole group was 300.04 ± 226.02 mg/cm^3^ (range − 88.68 to 555.06 mg/cm^3^) for region of interest (ROI 5) (central), 214.80 ± 167.45 mg/cm^3^ (range − 7.16 to 597.21 mg/cm^3^) for ROI 1–4 (marginal zones: medial, lateral, ventral, dorsal) and 168.14 ± 65.54 mg/cm^3^ (range 17.47–208.97 mg/cm^3^) for healthy bone tissue (femur and tibia).

**Conclusion:**

Based on WOMAC scores, LEFS, ambulatory status, and quality of life findings, it can be concluded that following tibial head ORIF with allograft bone patients has promising results.

## Introduction

Reconstruction of skeletal defects is a challenging problem in orthopaedic and trauma surgery [1–3]. Especially tibial plateau fractures can result in various degrees of tibial plateau depression. The resulting bone defects have to be reconstructed to restore the leg axis and regain the original function of the knee joint [4]. In these defects, human bone allografts and synthetic bone graft substitutes can be used as filling material [5, 6]. Bone allografts, as compared to synthetic bone grafts, have the advantage of possessing an osteoinductive (ability to induce new bone formation) and an osteoconductive (ability to facilitate new bone formation) potential [7].

Further, bone grafts can also be used as antibiotic carriers preventing bacterial infections directly at the surgical site [8, 9]. Bone grafting aims to restore the original anatomical conditions such as stability alignment, support an early range of motion and mobilization, and lower post-traumatic osteoarthritis risk [10–13]. Autograft bone, routinely obtained from the iliac crest, is commonly used to fill bone defects while facing the problem of restricted availability and the risk of infection, fracture, nerve injury, donor site pain, and hematoma [5, 10, 14–17].

There are few reports on patient outcome and bone union following tibial plateau fractures treated with bone allografts [4, 16, 18–20]. Previous studies have often assessed treatment outcomes using non-validated author-created scoring systems that relied heavily on physician-based assessments such as range of motion, knee stability, and radiograph [15, 21].

Therefore, we aimed to perform a 10-year follow-up investigation of patients treated with bone allograft during tibial head ORIF. For this purpose, participants’ radiological and clinical results were evaluated. We assumed (1) that the bone mineral density in the area of bone allograft treatment has no significant difference as compared to the measurements of healthy bone and (2) good clinical outcome as determined from the patient-reported outcome.

## Patients and methods

The local Institutional Review Board approved the study protocol (Registry Number: 1318/2019). The study was registered in a study trial register. Informed consent was signed and obtained from all patients before participation in our study. We identified all patients treated for a tibial plateau fracture with the additional use of bone allograft at the Department of Orthopaedics and Trauma Surgery of the Medical University of Innsbruck between 01.01.2010 and 15.04.2020. Data were collected from our internal patient register and the death register (Department of Clinical Epidemiology, Tirol Kliniken). We included all patients who had suffered a medial, lateral, or bicondylar fracture of the tibial plateau and undergone surgical treatment by open reduction and internal fixation (ORIF) using femoral head bone allografts.

The identified patients were contacted and invited to undergo the following examinations. Data such as patient age, follow-up time, body mass index (BMI), American Society of Anesthesiologists Score (ASA Score) [22], Charlson Comorbidity Index (CCI) [23], additional injuries, previous illnesses, and details of the surgery were collected from the patients’ medical records as well as by calling and inviting them to provide further information.

Fractures were classified according to the Schatzker Classification and the AO/OTA Classification (Arbeitsgemeinschaft für Osteosynthesefragen und Orthopedic Trauma, Association Committee for Coding and Classification) based on the preoperative radiographs [24–26].

Patient-reported clinical outcome was assessed using the “Western Ontario and McMaster Universities Osteoarthritis Index” (WOMAC) [27], a tested questionnaire for evaluation of symptoms and physical functional limitations in patients with osteoarthritis of the knee or hip. The WOMAC questionnaire consists of four scales, WOMAC pain, WOMAC stiffness, WOMAC function and WOMAC total (best value: 0, worst value: 100).

In addition to the limb-specific outcome, “health-related quality of life” was assessed using the EuroQol Group EQ-5D-3L health questionnaire [28]. The standard format and model (EQ-5D-3L) of the descriptive health classification system used in this study consists of five questions on the following health dimensions: mobility, self-care, activities of daily living, pain/discomfort, and anxiety/depression. There are three options for each of the five dimensions: no problems, some problems, extreme problems, which result in 243 potential health states. Two ratios—time trade-off (TTO) and visual analog scale (VAS)—were calculated from this dataset. These ratios are on a scale where full health is rated 1 and death 0 [29]. In addition, the questionnaire consists of the EQ-VAS. In this score, the patients were asked for a self-assessment of their current health state (5 = worst conceivable health state, 1 = best conceivable health state).

More intense activities such as running or jumping were assessed with the Lower Extremity Functional Scale (LEFS) [30]. The questionnaire consists of 20 items for assessing the functional status of a patient’s lower limb. There are five response options for each item: extreme difficulty/impossible (0 points), fairly great difficulty (1 point), moderate difficulty (2 points), little difficulty (3 points), no difficulty (4 points). The LEFS total score is obtained by summing the individual items and can vary from 0 to 80, with higher scores indicating higher lower extremity functional status [31, 32].

The Parker Mobility Score assessed ambulatory status. The Parker Mobility Score consists of three situations (able to get about the house, get out of the house, go shopping), each rated from 0 to 3 points. The total score ranges from 0 to 9, with 9 indicating the best possible mobility [33, 34].

**Fig. 1:**
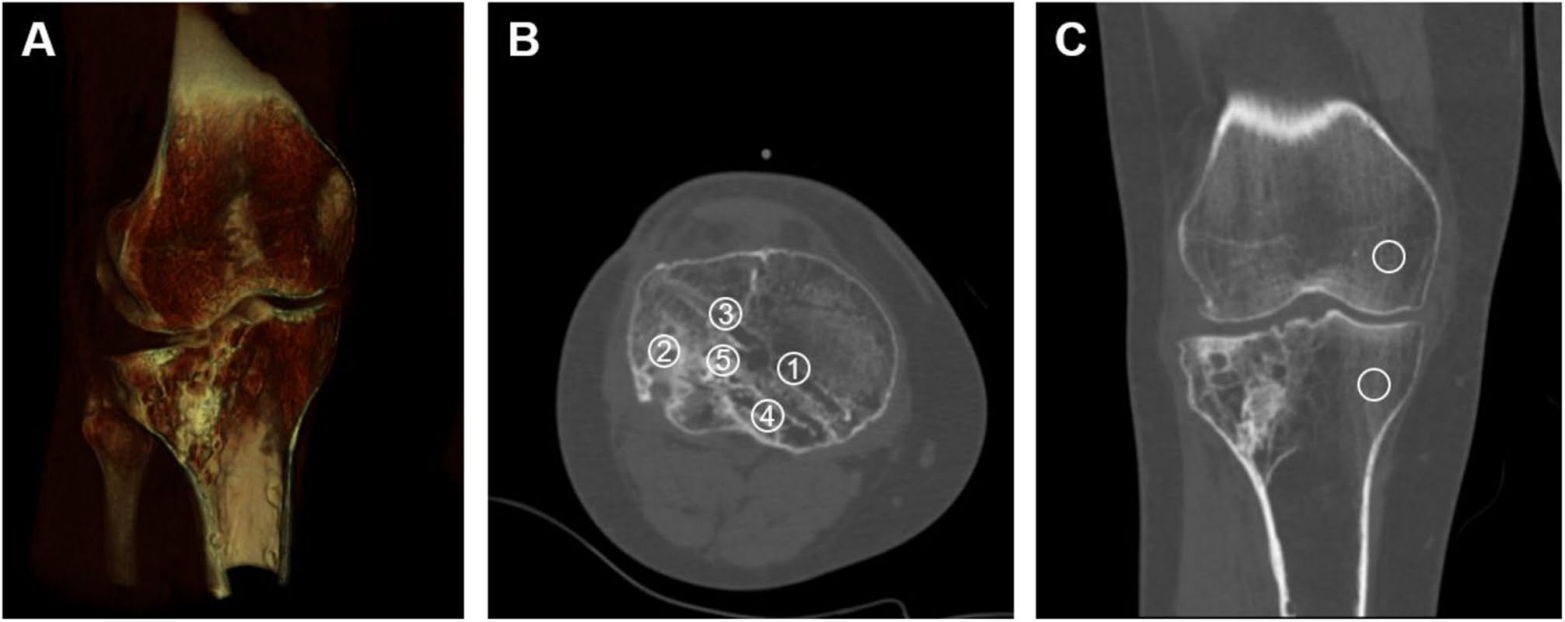
Example illustrating the placement and determination of bone mineral density using CT images. **A** 3D reconstruction of the right knee; **B** measurement of BMD of the marginal zones (ROI 1–4, medial, lateral, ventral, dorsal) and the central BMD (ROI5); **C** measurement of BMD of the healthy tissue of the femur and the tibia

CT scans were performed with a GE Discovery CT750 HD Scanner (GE, Milwaukee, WI, USA) using a standardized protocol (120 kV, mA Auto, 8.8 s/HE; 1.25 mm slice thickness) without radiopaque material, extending from the distal femoral shaft through the knee and proximal tibia. Quantitative computed tomography (QCT) was calculated from these CT images (QCT). Bone mineral density (BMD) was calculated from five circular regions of interest (ROI) of the grafted area: medial (1), lateral (2), ventral (3), dorsal (4) and central (5),using the PACS software Impax EE (Agfa HealthCare N.V., Mortsel, Belgium) (Fig. 1). Calculation of the BMD in mg/cm^3^ was performed with the European Forearm Phantom (EFP) as described in previous studies [35–39]. Each ROI had a diameter of 8.5 mm. In addition, the central BMD of adjacent “healthy” bone tissue (neither trauma nor surgery) of the tibia and femur was determined. The values of the marginal zones (ROI 2–5, medial, lateral, ventral, dorsal) and the values of the healthy tissue (tibia and femur) were averaged. It was reported that QCT exposes the patient to a minimal radiation dose when appendicular bones are investigated (< 1 µSv) [35].

Descriptive statistics were calculated as medians and means. Interquartile ranges (IQR) for numeric data and frequencies were used for ordinal data (medians) and ranges (min.–max.; means). Microsoft Excel 2020, version 14.36, (Microsoft Corporation, Redmond, Washington, USA) was used to analyze the data. Microsoft Excel 2020, version 14.36, and Prism, version 9.1.2, (Graphpad Software Inc, La Jolla, CA, USA) were used to create charts and display the data as tables and diagrams. Ordinal data are shown as absolute or relative frequencies. The median and interquartile range were chosen as descriptive statistics. The interquartile range was calculated using a weighted average. In the graphic representations of the medians (box plot, bar charts, line charts) the interquartile range was defined using Tuckey’s hinges. For Hypothesis 1, the unpaired *t* test was applied; *p* values < 0.05 were regarded as statistically significant.

## Results

**Fig. 2:**
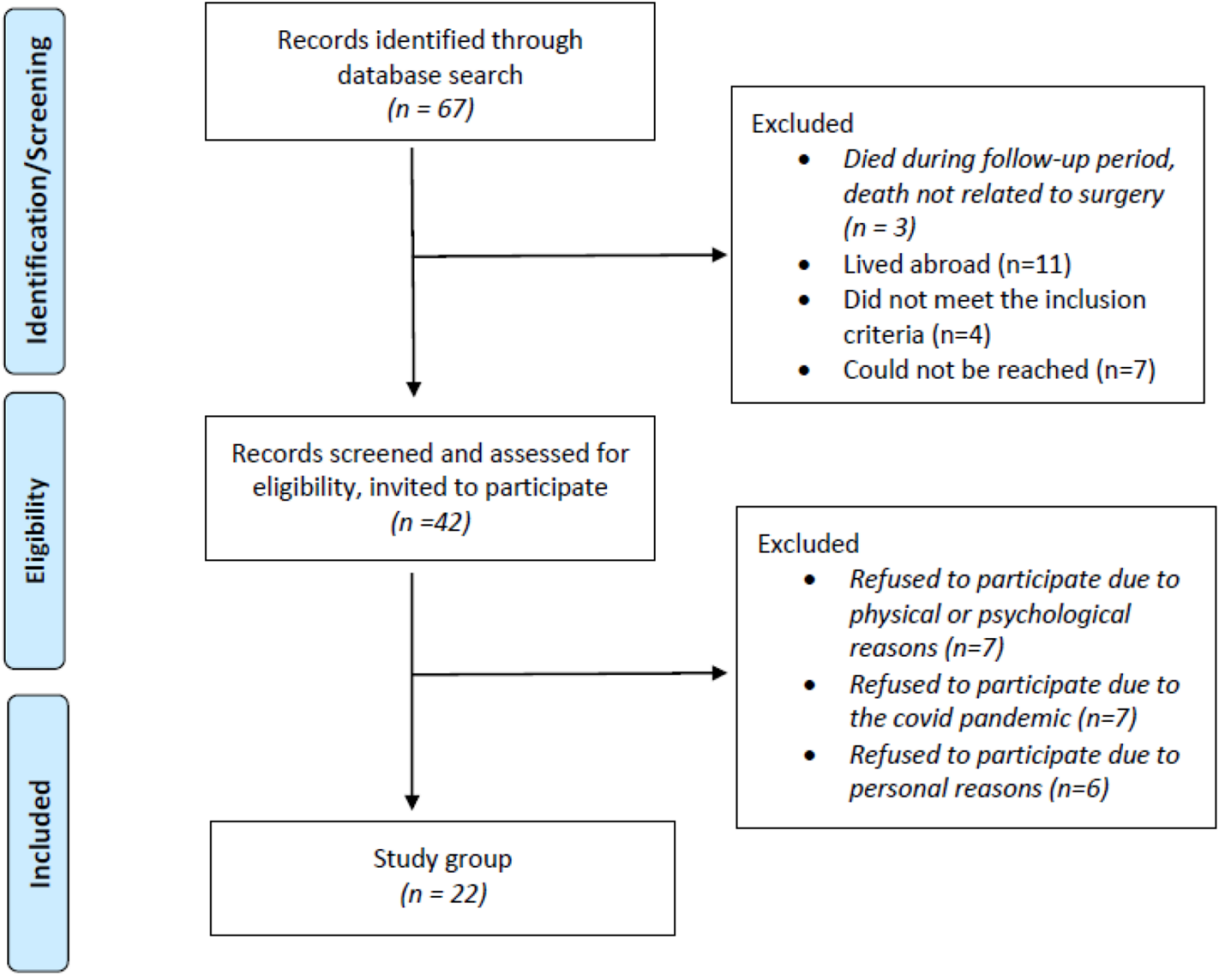
Flowchart for patient inclusion

In the period from 01.01.2010 to 15.04.2020, a total of 67 adults (> 18 years) with tibial plateau fracture undergoing surgery using bone allograft were found in the patient registry of the Medical University Hospital for Orthopaedics and Traumatology in Innsbruck. A total of 22 patients, 13 of whom were female and 9 male, with a mean age of 53.0 ± 13.8 (range 26–79) years and a mean body mass index of 23.8 ± 5.00 kg/m^2^ (range 17.4–51.2) were included in our study. Details are shown in the flowchart below (Fig. 2). Mean follow-up time was 2.88 ± 2.46 years (range 0.75–6.58 years).

At the time of surgery, mean CCI was 1.45 (range 0–4), mean ASA Score was 2.6 (range 1–3). Eleven cases involved a fracture of the lateral tibial condyle and one involved the medial tibial condyle. Additional surgery-related data are shown in the Appendix (Table 1). Fractures were classified according to the AO/OTA and the Schatzker Classification. The most common fractures observed in this study were classified as Schatzker II (11 patients 50.0%) or AO/OTA 41.B3 (12 patients 54.5%) fractures. Two (9.1%) patients had a Schatzker type IV, three (13.6%) patients a Schatzker type V and six (27.3%) patients a Schatzker type VI fracture. AO/OTA type 41.C1 was observed twice (9.1%), AO/OTA type 41.C2 once (4.6%) and AO.OTA 41.C3 seven times (31.8%).

**Table 1:** Surgery details

	Median, range
BMI (kg/m^2^)	23.8 (17.4–51.2)
ASA Score	2 (1–3)
Charlson comorbidity index	1.45 (0–4)
Follow-up (a)	2.88 (0.75–6.58)
Age at surgery (a)	53.0 (26–79)
Condyle	11 lateral
	1 medial
	10 bicondylar
Number of plates	1 (0–4) one patient received pin fixation
Tissue reconstruction	8 lateral menisci
	1 anterior cruciate ligament
External fixateur	10

WOMAC total was 13.0 (IQR = 16.3, range 0–33). The median for WOMAC pain was 7 (IQR = 13.5, range 0–32), for WOMAC stiffness 12.5 (IQR = 20.0, range 0–60), and for WOMAC function 11.5 (IQR = 15.8, range 0–35).

**Fig. 3:**
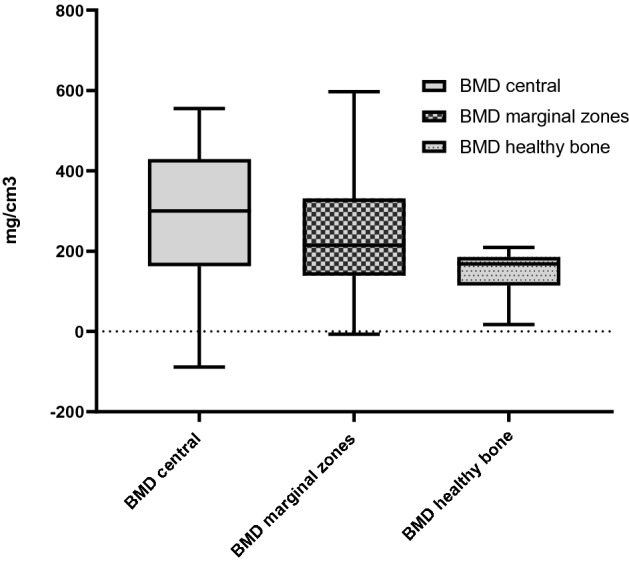
Presentation of the central BMD, the marginal zones BMD (mean value of the BMD of the medial, lateral, ventral, dorsal marginal zones) and the adjacent healthy bone (mean value of the BMD of the tibia and femur) for *n* = 22 using a box plot representation. The median ± interquartile range in mg/cm^3^ and the minimum and maximum measured values are shown

The median EQ-5D score for TTO was 0.887 ± 0.121 (range 0.361–1.000) and for VAS 0.794 ± 0.250 (range 0.381–1.000). Median LEFS score was 57.5 ± 19.0 (range 33–79). Mean Parker Mobility Score was 9 (range 6–9). All patients except one achieved the maximum score of 9. The 72-year-old female patient who did not achieve the maximum score reported a score of 6. Values are summarized in Table 2. In 19 cases, the bone allograft was visible in the plain radiographs. In the three remaining participants, the allograft tissue was completely resorbed. Median BMD for the whole group was 300.04 ± 226.02 mg/cm^3^ (range − 88.68–555.06 mg/cm^3^) for ROI 5 (central), 214.80 ± 167.45 mg/cm^3^ (range − 7.16–597.21 mg/cm^3^) for ROI 1–4 (marginal zones: medial, lateral, ventral, dorsal) and 168.14 ± 65.54 mg/cm^3^ (range 17.47–208.97 mg/cm^3^) for healthy bone tissue (femur and tibia). Details are shown in Table 2 and Fig. 3.

**Table 2:** Patient-reported and radiological outcome (detailed)

ID	WOMAC total (best: 0 worst: 100)	Parker Mobility Score (best: 9, worst: 0)	LEFS (best: 80, worst: 0)	EQ-5D TTO (best: 1, worst: 0)	EQ-5D VAS (best: 1, worst: 0)	BMD central	BMD marginal zones	BMD healthy bone
1	19	6	33	0.788	0.750	465.82	328.70	17.47
2	0	9	75	1.000	1.000	− 0.95	59.90	42.33
3	8	9	70	1.000	1.000	189.87	228.02	115.14
4	22	9	62	0.887	0.902	294.02	175.44	189.72
5	9	9	46	0.887	0.794	229.5	153.11	166.16
6	16	9	49	0.361	0.381	348.87	199.51	175.77
7	2	9	69	0.999	0.757	− 44.96	−7.16	126.03
8	18	9	54	0.887	0.649	− 88.68	75,28	63.90
9	6	9	66	1.000	1.000	409.82	235.40	185.14
10	20	9	41	0.788	0.750	394.05	501.10	208.97
11	19	9	64	0.887	0.902	306.05	203.62	205.09
12	0	9	75	1.000	1.000	199.07	168.23	173.92
13	17	9	46	1.000	1.000	219.59	225.98	148.26
14	4	9	55	0.887	0.649	344.25	311.23	186.89
15	1	9	77	1.000	1.000	80.37	97,81	170.13
16	33	9	56	0.877	0.794	453.92	367.77	171.95
17	10	9	50	0.877	0.794	555.06	424.07	112.90
18	17	9	55	0.887	0.649	71.52	69,17	139.72
19	27	9	36	0.788	0.750	530.32	597.21	64.46
20	1	9	79	1.000	1.000	291.24	186.19	178.17
21	17	9	59	0.887	0.649	420.976	338.38	200.87
22	0	9	63	1.000	1.000	483.9	249.06	165.43

Looking at bone mineral density as a function of follow-up time, it is noticeable that the BMD of the healthy tissue initially showed lower values than that of the graft (central ROI and ROIs of the marginal zones).

However, with a longer follow-up time, the graft values converged to the measured BMD values of the healthy bone matrix. It was found that the bone mineral density of the graft (central and marginal zones) after surgery was higher than the bone mineral density of the healthy tissue. With a longer follow-up time, BMD decreased to resemble that of the healthy bone tissue of the same patient.

## Discussion

The most important findings of our study were that patients had clinically successful results as shown by their patient-reported outcomes WOMAC, EQ-5D quality-of-life score,LEFS, and the Parker Mobility Score. Therefore, participants achieved better clinical outcomes and lower bone mineral densities with longer follow-up times.

Only few previous studies have investigated the patient-reported outcome of bone allografting after tibial plateau fractures [4, 6, 12, 16, 20]. Gausden et al. [12] retrospectively reviewed 61 patients with a mean age of 59.3 years and a mean follow-up of 59 months. In contrast to this study, only 64% (*n* = 39) patients were treated with bone void filler. Like our study, most of the fractures were classified as Schatzker II tibial plateau fractures. Age was negatively correlated with BMD measurements in healthy tissue. Schatzker et al. [40] mentioned poor bone quality as a significant obstacle for rigid fixation in tibial plateau fractures.

Ong et al. [6] analyzed 24 patients, of whom 7 were treated with bone allograft, 14 with synthetic bone, and 3 with autografts. The majority of the fractures were classified as Schatzker II tibial plateau fractures. Mean age in the bone graft group was 53 years. The WOMAC score after 12 months was 14.1 ± 6.03, which is in line with the results of our study.

Another study that compared clinical and radiological outcomes of tibial plateau fractures was performed by Bagherifard et al. [4] In that study, 58 patients were treated with bone allograft and achieved a Rasmussen clinical score of 18.45, which indicates good results, similar to our study. A positive correlation between patients’ clinical and radiological scores was observed. However, Bagherifard et al. [4] used Rasmussen radiological criteria in their study, namely articular depression, varus/valgus, and condylar widening factors.

Lasanianos et al. [20] presented data on 25 patients who received a freeze-dried cancellous allograft at a mean age of 53.2 years, mean follow-up of 13 months, and mostly Schatzker II fractures. The study also showed an excellent Rasmussen clinical score of 18.

Previous research shows only poor correlations between bone mineral density and patient-reported outcomes. While some authors of studies in which patients were treated without the use of bone allografts have reported an association between fracture pattern [41] or lower bone mineral density in non-injured bone-tissue [21, 42–44] and poor clinical outcome, our study and other recent literature [12] show that bone graft can address poor osseous integrity. The use of appropriate bone grafting and fixation technique can therefore mitigate the impact of poor bone quality on patient outcome and the influence of fracture severity. The previous studies show promising results in radiological and patient-reported outcomes of tibial plateau fractures treated with human bone allograft. Those results are highly congruent with the current study’s findings when comparing WOMAC scores, LEFS, good quality of life, good ambulatory status, and bone mineral density.

The following limitations of our study are acknowledged. First, limitations are due to the small sample size. Of 67 patients in our bone bank registry, only 42 were able to be invited to participate. Because of the ongoing Covid-19 pandemic, physical, psychological or personal reasons, 20 patients refused to participate, leaving only 22 patients for the study.

Another limitation is that the patients were operated on by a variety of surgeons at our institution. As longer follow-up has shown that the BMD in the allograft area adapts to the BMD of healthy bone, longer follow-up examinations should be conducted.

A strength of our study is the fact that all patients were assessed with clinically validated patient-reported outcomes. These questionnaires were compared with the CT scan measurement of the bone mineral density of the allograft bone.

From this study, it can be concluded that patients who undergo surgery after tibial plateau fracture by means of ORIF using bone allograft have promising results. The findings for WOMAC scores, LEFS, ambulatory status, and quality of life underline the method's success in this study. Therefore, the use of bone allograft is recommended for broader clinical use in the fixation of tibial plateau fractures, taking into account individual circumstances and an appropriate surgical procedure.

## Abbreviations

ORIF: Open reduction and internal fixation
WOMAC: Western Ontario and McMaster Universities Osteoarthritis Index
EQ-5D: EuroQol Five Dimension score
BMD: Bone mineral density
ROI: Region of interest
QCT: Quantitative computed tomography
BMI: Body mass index
ASA-Score: American Society of Anesthesiologists Score
CCI: Charlson Comorbidity Index
TTO: Time trade-off
VAS: Visual analog scale
